# Serological Evidence of Avian Influenza in Captive Wild Birds in a Zoo and Two Safari Parks in Bangladesh

**DOI:** 10.3390/vetsci7030122

**Published:** 2020-09-01

**Authors:** Mohammad M. Hassan, Mohamed E. El Zowalaty, Ariful Islam, Md. M. Rahman, Md. N. U. Chowdhury, Hatem S. M. Z. Nine, Md. K. Rahman, Josef D. Järhult, Md. A. Hoque

**Affiliations:** 1 Faculty of Veterinary Medicine, Chattogram Veterinary and Animal Sciences University, Khulshi, Chattogram 4225, Bangladesh; arif@ecohealthalliance.org (A.I.); kaisarrahman@ecohealthalliance.org (M.K.R.); md.hoque@my.jcu.edu.au (M.A.H.); 2 Department of Clinical Sciences, College of Medicine, University of Sharjah, Sharjah 27272, UAE; 3 Sharjah Institute of Medical Research, College of Medicine, University of Sharjah, Sharjah 27272, UAE; 4 Department of Medical Biochemistry and Microbiology, Zoonosis Science Center, Uppsala University, SE-75 123 Uppsala, Sweden; 5 Centre for Integrative Ecology, School of Life and Environmental Science, Deakin University, Geelong Campus, Geelong VIC 3216, Australia; 6 EcoHealth Alliance, New York, NY 10001-2320, USA; 7 Bhanghabandhu Sheikh Mujib Safari Park, Cox’s Bazar 4740, Bangladesh; mmustafizsafari@gmail.com; 8 Sheikh Kamal Wildlife Center, Gazipur 1740, Bangladesh; nizamvet05@gmail.com; 9 Bhanghabandhu Sheikh Mujib Safari Park, Gazipur 1740, Bangladesh; hatemdvm@gmail.com; 10 Department of Medical Sciences, Zoonosis Science Center, Uppsala University, SE-751 85 Uppsala, Sweden; Josef.Jarhult@medsci.uu.se

**Keywords:** avian influenza, zoonotic, surveillance, sero-prevalence, AIV antibodies, c-ELISA, real-time RT-PCR, H9 subtype, captive wild birds, zoo, safari park

## Abstract

Avian influenza (AI) is endemic and frequently causes seasonal outbreaks in winter in Bangladesh due to high pathogenic avian influenza (HPAI) H5N1 and low pathogenic avian influenza (LPAI) H9N2. Among avian influenza A viruses (AIV), H5, H7, and H9 subtypes have the most zoonotic potential. Captive birds in zoos and safari parks are used for educational, recreational, breeding, and conservational purposes in Bangladesh. To screen for AIV in captive birds to assess potential public health threats, we conducted a cross-sectional study in two safari parks and one zoo in Bangladesh for four months, from November to December 2013 and from January to February 2014. We collected blood samples, oropharyngeal, and cloacal swabs from 228 birds. We tested serum samples for AIV antibodies using competitive enzyme-linked immunosorbent assay (c-ELISA) and AIV sero-subtype H5, H7, and H9 using hemagglutination inhibition (HI) test. Swab samples were tested for the presence of avian influenza viral RNA using real-time reverse transcription-polymerase chain reaction (rRT-PCR). Across all the samples, AIV antibody prevalence was 9.7% (95% CI: 6.1–14.2, n = 228) and AIV HA subtype H5, H7 and H9 sero-prevalence was 0% (95% CI: 0–1.6, n = 228), 0% (95% CI: 0–1.6, n = 228) and 6.6% (95% CI: 3.72–10.6, n = 228), respectively. No AI viral RNA (M-gene) was detected in any swab sample (0%, 95% CI: 0–1.6, n = 228). Birds in the Safari park at Cox’s Bazar had a higher prevalence in both AIV antibody prevalence (13.5%) and AIV H9 sero-prevalence (9.6%) than any of the other sites, although the difference was not statistically significant. Among eight species of birds, Emu (*Dromaius novaehollandiae*) had the highest sero-positivity for both AIV antibody prevalence (26.1%) and AIV H9 prevalence (17.4%) followed by Golden pheasant (*Chrysolophus pictus*) with AIV antibody prevalence of 18.2% and AIV H9 prevalence of 11.4%. Our results highlight the presence of AI antibodies indicating low pathogenic AIV mingling in captive birds in zoos and safari parks in Bangladesh. Continuous programmed surveillance is therefore recommended to help better understand the diversity of AIVs and provide a clear picture of AI in captive wild birds, enabling interventions to reduce the risk of AIV transmission to humans.

## 1. Introduction

Avian influenza (AI) is a major viral infectious disease affecting both avian species and humans, caused by influenza A virus and circulating throughout the globe [1]. Avian influenza A viruses are classified as high pathogenic avian influenza (HPAI) or low pathogenic avian influenza (LPAI). Wild waterfowls are the natural reservoir of influenza A viruses which are responsible for the circulation of these viruses into domesticated poultry and other captive birds. By exposure to affected poultry, humans can then be infected with avian influenza viruses (AIVs) [2]. The devastating HPAI H5N1 virus was transmitted from birds to humans in live bird market (LBM) in Hong Kong in 1997 [3]. AI H9N2 virus subtype is considered as LPAI and was first reported in China in 1992 [4], and in South Korea, Iran, and Pakistan [5,6,7] in 1996, 1998 and 1999, respectively. Since 2008, various LPAI subtypes were identified from different farms in different parts of Bangladesh [8]. Despite using sporadic vaccination, H5N1 subtype was also detected in farms in Bangladesh [9,10]. A recent study from Bangladesh reported that wild birds of the order *Anseriformes* are the main reservoir of H5N1, and the house crow (*Corvus splendens*) under the order of *Passeriformes* which are significantly living on offal from LBMs showed higher AI sero-prevalence [11,12]. Both LPAI and HPAI co-infections are also circulating in LBMs, poultry farms, and backyard chicken all year round, which is of significant concern [13].

Zoos and safari parks in Bangladesh are important tourism venues, and they contain a variety of captive and wild bird species. Zoos are also a focused exhibition center for different wild animals and birds [14]. Wild captive bird species are kept separately in cages, however AIV can be transmitted through various ways between cages from birds to visitors. The cages of the birds are placed in close proximity to visitors in most of the places which potentially enabling the transmission of AIVs from birds to humans. Moreover, the cleaning waste of the cages are deposited in the nearby lowlands, which is also a potential bio-risk. It was previously reported that HPAI H5N1 outbreaks occurred in wild and captive birds (carnivores) in Cambodia due to virus-infected poultry used as feed [15]. Furthermore, an HPAI H5N1 outbreak occurred in captive birds in the Phnom Tamao Wildlife Rescue Centre, in Cambodia [15]. It was previously reported that 1.6% of birds were seropositive for AI H5 subtype in a zoo in the Netherlands [16]. Migratory birds are the possible source of AIV infection in zoos and safari parks in different parts of the world [17,18,19], since each of these establishments have small attached lakes and wetlands. Seasonal migratory birds commonly harbor AIVs, which then probably are transmitted to captive wild birds through fomites, water or air-borne. Strict bio-security measures have been implemented to prevent captive wild birds from mixing with migratory birds during the migratory seasons [20,21]. Considering the above facts, we screened AIVs in captive wild birds in one zoo and two safari parks in Bangladesh to assess the prevalence of AIV among bird species and the potential public health threats. This is the first study in Bangladesh to perform AIV surveillance using molecular methods of PCR and serology in captive wild birds in zoos and safari parks.

## 2. Materials and Methods

### 2.1. Study Location

**Figure 1:**
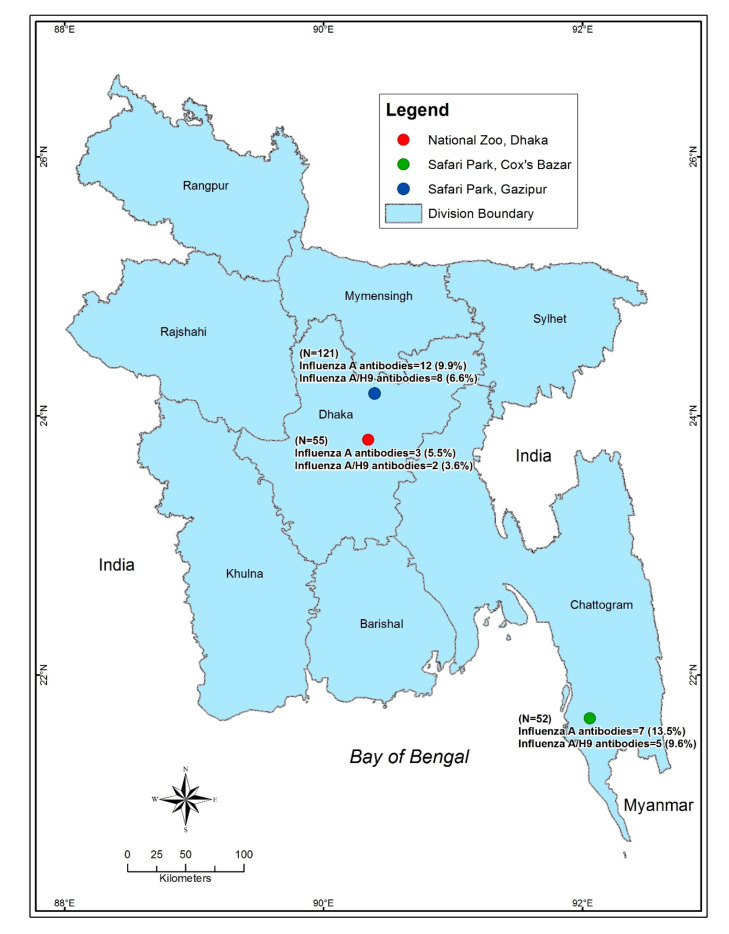
Map of Bangladesh showing the sampling locations. The map was plotted using the spatial analyst tool of ArcGIS (ArcMap, version 10.2, Environmental Systems Research Institute, Redlands, California, CA, USA). The three dots depict the sampling locations (Zoo and safari parks). The green dot depicts the Safari park of Cox’s Bazar, the red dot depicts the National zoo, Dhaka and the Blue dot depicts the Safari park at Gazipur.

We sampled a wide range of captive wild birds from the National Zoo, Dhaka, Bangabandhu Sheikh Mujib Safari park, Gazipur, and Bangabandhu Sheikh Mujib Safari park, Cox’s Bazar (Figure 1, Supplementary Table S1), between November 2013 and February 2014. Highly pathogenic avian influenza outbreaks usually occur in the winter months (November–February) in Bangladesh [22]. We sampled a total of 228 birds from 8 different species of captive wild birds including 23 Emu (*Dromaius novaehollandiae*), 44 Golden pheasants (*Chrysolophus pictus*), 32 Green peafowls (*Pavo muticus*), 37 Guinea fowl (*Numida meleagris*), 30 Macaw (*Ara macao*), 17 Ostrich (*Struthio camelus*), 22 Raj Donesh (*Buceros bicornis*), and 23 Red napped ibis (*Pseudibis papillosa*).

### 2.2. Ethical Approval

Captive wild birds capture was performed and approved by the Bangladesh Forest Department, The Peoples Republic of Bangladesh (permit reference number: WASU/FAO/PSWMID-6/2012/58). Handling and sampling of birds were approved by the Chattogram Veterinary and Animal Sciences University Animal Experimentation Ethics Committee (permit ref. no. CVASU/Dir (R and E) AEEC/2015/02), Bangladesh. All birds were released without injury or harm into their cages after sampling, and all procedures were made to minimize animal suffering throughout the research study.

### 2.3. Sample Collection

Cloacal and oro-pharyngeal swabs were collected from captive wild birds, along with blood samples from each bird. Swab samples were obtained from birds by inserting swab sticks into the vent (until fecal contamination) for cloacal swabs and oro-pharyngeal airway and the wall of the oro-pharynx for oro-pharyngeal swabs. Each of the cloacal and oropharyngeal swab samples was placed independently into a vial containing one ml of sterile viral transport medium as previously described [23]. Samples were stored immediately in a dry shipper with liquid nitrogen after collection until transferred to −80 °C storage in the Poultry Research and Training Centre (PRTC) laboratory at Chattogram Veterinary and Animal Sciences University (CVASU), Bangladesh.

### 2.4. Competitive Enzyme-Linked Immunosorbent Assay (c-ELISA)

Whole blood samples were collected (0.5–3 mL in all cases < 1% of body weight) aseptically from wing veins or jugular veins and then immediately transferred to individual sterile tubes labeled with unique identity numbers. Blood samples were subsequently allowed to clot at ambient temperature, kept refrigerated overnight, followed by centrifugation at 10,000 rpm for 30 min at 4 °C to collect serum samples. Serum samples were then transferred into cryo-vials and kept at −20 °C [24]. Serum samples were tested using competitive enzyme-linked immunosorbent assay (c-ELISA) according to the manufacturer’s protocol [25]. In c-ELISA, avian influenza antibodies were detected against AI nucleoprotein (NP) using commercial test kit ID.vet ID Screen^®®^ (Catalog No. FLUACAver 1216 GB; Sensitivity 100% and Specificity 96%; ID.vet, Garbles, France). The specific antigen-antibody binding was detected and measured. The optical density (OD) of the final plate was recorded at 450 nm. The results were interpreted as per the manufacturer protocol, and the signal to noise ratios (the quotient of the sample mean absorbance divided by negative control mean absorbance) of 0.45 or less was considered positive.

### 2.5. Hemagglutination Inhibition (HI) Test

HI test was performed for antibodies against AIV subtypes H5, H7 and H9 using the c-ELISA reactive serum samples using specific antigen according to the protocol by the Australian Animal Health Laboratory (AAHL) (East Geelong, VIC 3220, Australia) [26]. Serum samples showing inhibition at dilutions of 1:16 or greater against four hemagglutination units of AIV antigen were considered reactive for the antibody of wild birds [27].

### 2.6. Real-Time Reverse Transcription-Polymerase Chain Reaction (rRT-PCR)

For molecular testing, individual cloacal and oropharyngeal swab samples of captive wild birds were subjected to RNA extraction using the MagMAXTM-96 Viral RNA Isolation Kit (ThermoFisher Scientific, Waltham, MA, USA) according to the manufacturer’s protocol (AM1836 and AMB1836-5). RNA extracts of cloacal and oropharyngeal swab samples were tested for AI viral RNA using rRT-PCR (Sensitivity: 99.5% and Specificity: 88.2%) using influenza A virus matrix (M) gene specific primers and probe to using the AgPath-ID One-Step RT-PCR kit (ThermoFisher Scientific, Waltham, MA, USA) (Catalog number AM1005) as previously described (H5 inactivated antigen was used as positive control in rRT-PCR reaction) [27,28].

### 2.7. Statistical Analysis

Microsoft Office Excel 2013 was used for data management and STATA/IC-13 (StataCorp, 4905, Lake Way Drive, College Station, TX 77845, USA) was used for data analysis. Descriptive analysis was performed to determine the positive percentage of the test results of different variables. The prevalence of AI was calculated as the number of birds with sero-positive or RNA positive or sero-subtype specific positive out of the total number of birds tested. Birds tested negative in c-ELISA and negative in subtype specific HI testing were considered as denominator when we calculated the subtype specific sero-prevalence and its distribution. Fisher’s exact test was performed for different variables (year, month, sites, and species) to identify the association between the response variable (test result, yes/no) and each of the selected explanatory variables. The results were expressed as a percentage, frequency number and 95% confidence interval (CI) and *p*-value (≤0.05 as significant).

## 3. Results

The overall prevalence of avian influenza antibodies was 9.7% (95% CI: 6.1–14.2, n = 228) in all captive birds (Supplementary Table S1). Most factors were not significantly associated with AI sero-prevalence. In year 2013, prevalence of AIV antibody was higher (13.3%, 95% CI: 6.6–23.2; n = 75) than the following year 2014 (7.8%, 95% CI: 4.1–13.3; n = 153). The winter month December showed the highest percentage of AIV antibody (21.4%, 95% CI: 4.7–50.8, n = 14). The sero-prevalence of AIV was higher in Safari Park (Cox’s Bazar) (13.5%, 95% CI: 5.6–25.8, n = 52) followed by Safari Park (Gazipur) (9.9%; 95% CI: 5.2–16.7, n = 12) and National zoo (Dhaka) (5.5%; 95% CI: 1.1–15.1, n = 3). Emu exhibited higher prevalence for AIV antibody (26.1%, 95% CI: 10.2–48.4) followed by Golden pheasant (18.2%; 95% CI: 8.2–32.7, n = 8), Guinea fowl (10.8%; 95% CI: 3.0–25.4, n = 4), Red-napped ibis (8.7%; 95% CI: 1.1–28.1, n = 2), Raj dhonesh (4.6%; 95% CI: 0.1–22.8, n = 1) and Green peafowl was lowest (3.1%; 95% CI: 0.07–16.2, n = 1) (Table 1). AI viral RNA (M-gene) was not detected (0%, 95% CI: 0–1.6, n = 228) by rRT-PCR in any of the tested swab samples in 8 species of birds from the zoo and the two safari parks of Bangladesh.

The sero-prevalence of AI H5, H7 and H9 subtypes in the captive bird in the present study was 0% (95% CI: 0–1.6), 0% (95% CI: 0–1.6) and 6.6% (95% CI: 3.72–10.6), respectively. None of the factors was significantly associated with the H9 sero-prevalence (Table 2). However, the H9 sero-prevalence was higher in 2013 (9.3%, 95% CI: 3.8–18.3; n = 7, in Safari parks (Cox’s Bazar) (9.6%, 95% CI: 3.2–21.0) or Gazipur (6.6%; 95% I: 2.9–12.6) than that of the counterpart part of each factor. Emu birds had higher positivity for AIV H9 (17.4%, 95% CI: 4.9–38.8; n = 23) than any of the bird species tested (Table 2).

## 4. Discussion

AI is a major viral infectious disease of concern in birds including poultry, resident, migratory and captive wild birds in Bangladesh. Safari parks and zoos of Bangladesh are used for education, recreation, animal breeding and conservation purposes. In these places, mass gatherings of people take place every day. There are 38 different species (n = 730), 25 species (n = 175) and 57 species of birds (n = 1155) in Bangabandhu Sheik Mujbur Rahman Safari park, Gazipur; Bangabandhu Sheik Mujbur Rahman Safari park, Cox’s Bazar; and the National Zoo, Dhaka, respectively (personal communication). Moreover, 2500, 1500, and 4000 persons per day with the highest numbers in winter (December, January, and February) visited Gazipur safari park, Cox’s Bazar Safari park and Zoo, Dhaka, respectively (personal communication). Therefore, there is a risk of transmission of zoonotic infectious diseases including AI from these captive birds to humans due to close contact. The current study investigated the presence of avian influenza among captive birds in one zoo and two safari parks using serological methods and molecular methods of RT-PCR. Serological testing demonstrated AIV antibodies and AIV antibodies of subtype H9, however no AI viral RNA was detected. These findings are similar to reports in captive wild birds in Nigeria (10.4% sero-prevalence) [29] and Ohio zoos (2006 to 2009, 11.9%) [30], in Little Egrets and Black-crowned Night Herons at a city park in Jiangxi, China (6.0% H9) [31]. A previous study in Bangladesh reported the detection of AIV H5 subtype in resident and migratory wild birds, which might be due to the possibility of mixing of different bird species in wild condition [32]. The absence of H5 and H7 in HI testing along with the absence of AI viral RNA in the present study is a positive sign since AI H5N1 virus infection causes huge mortality in domestic and captive birds in different parts of the world [15]. These results are supported by a number of studies from different parts of the world such as in Iran in 2015 [33] and two zoos in Delhi and Madhya Pradesh in 2016 [34]. Moreover, some birds which are naturally pre-exposed to LPAIV can play a role as AIV reservoir and play a role in virus transmission to susceptible host species and sometimes have homologous immunity that prevent the transmission [35].

In November and December (winter months), there was an increased AI sero-prevalence in the present study, which might be related to the coincidence of human influenza A virus ofH1 and H3 subtypes reported in humans in the winter season in Bangladesh [36]. Moreover, during the winter season, there was a report of house crow (*Corvus splendens*) died due to HPAI H5N1 in Bangladesh [11,37] and a report of high AI sero-prevalence and the subtypes H5 and H9 in crow, house sparrow, and common myna in Bangladesh [11,38]. Therefore, the house crow or other resident wild birds [32] might play a role in infection of captive wild birds in zoo and safari park. In the present study, AIV H9 subtype prevalence was higher in Emu than that in other captive wild birds which corroborates findings in several previous studies [30,32], for instance 28.2% Emu’s were positive for antibodies against AI H9N2 by HI in Maharashtra state, India, during the period from 2010 to 2011 [39].

The presence of AI antibody in Golden pheasants was the second highest in the current study, which is similar to other studies [40,41]. We did not detect AIV sero-positivity in Macaws and Ostriches. We recommend further AIV sampling and testing in zoos and safari parks throughout the entire year. Limitations of the present study include the small sample number and the lack of power calculations (sampling done without statistical assumptions), convenience sampling (as there was no ringing or banding system to identify the captured birds) and the lack of bird demographic information.

## 5. Conclusions

Captive wild birds in zoos and safari parks are used for educational, recreational, breeding, and conservational purposes, thus constituting a potential zoonotic hazard. We demonstrated AIV antibody prevalence (9.7%) and H9 sero-prevalence (6.6%), while AI viral RNA, H5, and H7 AIV antibodies were not detected. Birds in the Safari park at Cox’s Bazar had non-significant higher prevalence of both AIV antibody and H9 sero-subtype than the other two sites. Emu was the species with the highest prevalence of AIV antibody and H9 subtype. We recommend the implementation of programmed AI surveillance in captive wild birds to be able to mitigate the risk of AIV transmission to humans.

## Figures and Tables

**Table 1:** Univariate association between different factors and avian influenza A viruses (AIV) antibody positive samples (n = 228).

Variable	Categories (N)	n (%)	95% Confidence Interval (CI)	*p*
Year	2013 (75)	10 (13.3)	6.6–23.2	0.187
	2014 (153)	12 (7.8)	4.1–13.3	
Month	January (23)	1 (4.4)	0.1–21.9	0.332
	February (130)	11 (8.5)	4.3–14.6	
	November (61)	7 (11.5)	4.7–22.2	
	December (14)	3 (21.4)	4.7–50.8	
Site	National Zoo, Dhaka (55)	3 (5.5)	1.1–15.1	0.370
	Safari Park, Cox’s Bazar (52)	7 (13.5)	5.6–25.8	
	Safari Park, Gazipur (121)	12 (9.9)	5.2–16.7	
Species	Emu (23)	6 (26.1)	10.2–48.4	0.011
	Golden pheasant (44)	8 (18.2)	8.2–32.7	
	Green peafowl (32)	1 (3.1)	0.07–16.2	
	Guinea fowl (37)	4 (10.8)	3.0–25.4	
	Macaw (30)	-	-	
	Ostrich (17)	-	-	
	Raj dhonesh (22)	1 (4.6)	0.1–22.8	
	Red-naped ibis (23)	2 (8.7)	1.1–28.1	

**Table 2:** Univariate association between different factors and AIV HA subtype H9 (n = 228).

Variable	Categories (N)	n (%)	95% Confidence Interval (CI)	*p*
Year	2013 (75)	7 (9.3)	3.8–18.3	0.24
	2014 (153)	8 (5.2)	2.3–10.0	
Month	January (23)	1 (4.4)	0.1–21.9	0.138
	February (130)	7 (5.4)	2.2–10.8	
	November (61)	4 (6.6)	1.8–15.9	
	December (14)	3 (21.4)	4.6–50.8	
Site	National Zoo, Dhaka (55)	2 (3.6)	0.4–12.5	0.460
	Safari Park, Cox’s Bazar (52)	5 (9.6)	3.2–21.0	
	Safari Park, Gazipur (121)	8 (6.6)	2.9–12.6	
Species	Emu (23)	4 (17.4)	4.9–38.8	0.107
	Golden pheasant (44)	5 (11.4)	3.8–24.6	
	Green peafowl (32)	1 (3.1)	0.07–16.2	
	Guinea fowl (37)	3 (8.1)	1.7–21.9	
	Macaw (30)	-	-	
	Ostrich (17)	-	-	
	Raj dhonesh (22)	-	-	
	Red-naped ibis (23)	2 (8.7)	1.1–28.0	

## Supplementary Materials

The following are available online at https://www.mdpi.com/2306-7381/7/3/122/s1, Table S1: Metadata of bird samples during avian influenza surveillance in 2013-2014 from zoo and safari parks in Bangladesh.
